# The anti‐parasitic drug miltefosine suppresses activation of human eosinophils and ameliorates allergic inflammation in mice

**DOI:** 10.1111/bph.15368

**Published:** 2021-02-02

**Authors:** Eva Knuplez, Melanie Kienzl, Athina Trakaki, Rudolf Schicho, Akos Heinemann, Eva M. Sturm, Gunther Marsche

**Affiliations:** ^1^ Division of Pharmacology, Otto Loewi Research Center Medical University of Graz Graz Austria; ^2^ BioTechMed‐Graz Graz Austria

**Keywords:** allergic inflammation, eosinophils, miltefosine

## Abstract

**Background and Purpose:**

Miltefosine is an alkylphosphocholine drug with proven effectiveness against various types of parasites and cancer cells. Miltefosine is not only able to induce direct parasite killing but also modulates host immunity, for example by reducing the severity of allergies in patients. To date, there are no reports on the effect of miltefosine on eosinophils, central effector cells involved in allergic inflammation.

**Experimental Approach:**

We tested the effect of miltefosine on the activation of human eosinophils and their effector responses *in vitro* and in mouse models of eosinophilic migration and ovalbumin‐induced allergic lung inflammation.

**Key Results:**

The addition of miltefosine suppressed several eosinophilic effector reactions such as CD11b up‐regulation, degranulation, chemotaxis and downstream signalling. Miltefosine significantly reduced the infiltration of immune cells into the respiratory tract of mice in an allergic cell recruitment model. Finally, in a model of allergic inflammation, treatment with miltefosine resulted in an improvement of lung function parameters.

**Conclusion and Implications:**

Our observations suggest a strong modulatory activity of miltefosine in the regulation of eosinophilic inflammation *in vitro* and *in vivo*. Our data underline the potential efficacy of miltefosine in the treatment of allergic diseases and other eosinophil‐associated disorders and may raise important questions regarding the immunomodulatory effect of miltefosine in patients treated for leishmania infections.

**Abbreviations**
BMDE: bone marrow‐derived eosinophil
fMLP: *N*‐formylmethionyl‐leucyl‐phenylalanine
FSC: forward scatter
PI: propidium iodide

**What is already known**

- Miltefosine is an orphan drug marketed for the treatment of leishmaniasis.
- Miltefosine reduces the severity of allergies in patients.

**What this study adds**

- Miltefosine inhibits activation of human eosinophils and suppresses human eosinophil effector responses.
- Miltefosine inhibits the infiltration of immune cells in the airways and improves animal lung function.

**What is the clinical significance**

- Miltefosine may serve as a potential candidate for the treatment of eosinophil‐related diseases.
- Miltefosine treatment may influence eosinophil host responses in leishmania‐infected patients.

## 1 INTRODUCTION

To date, miltefosine (Impavido®) is the only oral drug approved for the treatment of leishmaniasis with limited mild or moderate side effects (Pijpers et al., 2019). The development of miltefosine is a success story of public–private partnership, a breakthrough in medicine affordability and patient drug adherence, landing it on the World Health Organization (WHO)'s List of Essential Medicines (Berger et al., 2017; Sunyoto et al., 2018). Miltefosine disrupts membrane structures and affects phosphatidylcholine synthesis in susceptible promastigote cells (Pinto‐Martinez et al., 2018; Rakotomanga et al., 2007). Due to its detergent‐like properties, miltefosine is thought to interact with the mucosa of the gastrointestinal tract during oral use and cause its most commonly listed side effects—nausea, vomiting and diarrhoea (Bhattacharya et al., 2007). During prolonged treatment, the severity of the side effects was reported to decrease over time (8.2% during Week 1 to 3.2% during Week 4) (Bhattacharya et al., 2007).

Miltefosine exerts immunomodulatory effects on human cancer cells by inhibiting the PI3K/Akt signalling pathway (Ruiter et al., 2003), induces IL‐12‐dependent Th1 responses (Wadhone et al., 2009) and shows anti‐inflammatory effects in endothelial cells, suppressing vascular inflammation (Fang et al., 2019). However, the immunomodulatory effects of miltefosine on primary human cells have so far only been described for T cells (Bäumer et al., 2010) and mast cells (Weller et al., 2009).

In plasma, miltefosine is mainly bound to albumin (96–98%) (Kip et al., 2018) and accumulates predominantly in cholesterol‐rich microdomains of the cell membranes (lipid rafts) (Malta de Sá et al., 2015). Miltefosine increases membrane fluidity (Moreira et al., 2014), modulates lipid raft‐dependent signalling (Weller et al., 2009) and could therefore be an attractive drug candidate for the treatment of diseases characterized by abundant lipid raft activation, such as allergic diseases (Dölle et al., 2010). Miltefosine attenuates allergic inflammation in T cell‐dependent mouse models of dermal inflammation (Bäumer et al., 2010), improves local dermatitis in patients with atopic dermatitis (Dölle et al., 2010), inhibits activation and degranulation of mast cells, and significantly reduces allergic disease manifestation in patients (Magerl et al., 2013; Maurer et al., 2013; Rubíková et al., 2018).

Surprisingly, there are no reports on the effects of miltefosine on eosinophils, a key cell type involved in the initiation and propagation of immune responses in allergic diseases (Stone et al., 2010). Here, we studied in detail whether miltefosine exerts immunomodulatory effects on eosinophils *in vitro* and in mouse models of allergic lung inflammation.

## 2 METHODS

### 2.1 Materials

Unless otherwise indicated, all purchased reagents were from Sigma (Vienna, Austria). Eotaxin‐2 (CCL24) used for *in vivo* chemotaxis and recombinant human C5a were acquired from R&D Systems (Minneapolis, MN, USA). Eotaxin‐1 (CCL11) and eotaxin‐2 (CCL24) used *in vitro* assays were obtained from ImmunoTools (Friesoythe, Germany). Antibody against phospho‐Akt (Ser 473) (Cat#9271, RRID:AB_329825) was obtained from Cell Signaling Technology (Danvers, MA, USA), while secondary goat anti‐rabbit Alexa Fluor 488 IgG antibody (Cat# A‐11008, RRID:AB_143165) was from Life Technologies (Thermo Fisher Scientific, Waltham, MA, USA). Annexin V, propidium iodide (PI) (Cat# 556547), CD63‐FITC (Cat# 561924, RRID:AB_10894192), Siglec‐F‐PE (Cat# 552126, RRID:AB_394341), CD3‐PE Cy5 (Cat# 553065, RRID:AB_394598), CD11b‐PE‐Cy7 (Cat# 552850, RRID:AB_394491) and CD11c‐BV421 (Cat# 562782, RRID:AB_2737789) were from BD Biosciences (Vienna, Austria). TruStain fcX CD16/32 (Cat# 101320, RRID:AB_1574975), Ly‐6C‐FITC (Cat# 128005, RRID:AB_1186134), Ly‐6G‐APC (Cat# 127613, RRID:AB_1877163), CCR3‐BV421 (Cat#144517, RRID:AB_2565743) and I‐A/I‐E‐V510 (Cat# 107635, RRID:AB_2561397) were from BioLegend (San Diego, CA, USA). Aluminium hydroxide gel used as an adjuvant was acquired from InvivoGen (Toulouse, France). CD11b‐FITC mouse anti‐human antibody (Cat# IMO53OU) used for measuring CD11b up‐regulation was obtained from Beckman Coulter (Krefeld, Germany). Miltefosine used in *in vivo* assays was purchased from Cayman Chemical (Ann Arbor, MI, USA). All functional assays of eosinophils were performed in assay buffer (PBS with Ca^2+^ and Mg^2+^, HEPES 10 mM, glucose 10 mM and bovine serum albumin 0.1%, pH 7.4).

Fixative solution was prepared by adding 9 ml of distilled water and 30 ml of FACS sheath fluid (BD Biosciences) to 1 ml of CellFix (BD Biosciences, Vienna, Austria) as described previously (Knuplez, Curcic, et al., 2020).

### 2.2 Mice

Animal studies are reported in compliance with the ARRIVE guidelines (Percie du Sert et al., 2020) and with the recommendations made by the *British Journal of Pharmacology* (Lilley et al., 2020). All animal experiments were performed in the animal facilities of the Medical University of Graz. The experimental procedure used in this study was approved by the Austrian Federal Ministry of Science, Research and Economy (protocol number: BMWFW‐66.010/0207‐WF/V/3b/2017); it conforms to Directive 2010/63/EU and was performed in accordance with national and international guidelines. BALB/c mice (RRID:IMSR_CRL:028) were either bred in‐house or obtained from Charles River. Δdbl GATA‐1 and interleukin‐5 (IL‐5) transgenic (IL‐5Tg) mice (both BALB/c background) were initially obtained from Dr Helene Rosenberg (NIH, Bethesda, MD, USA) and bred in our facilities. IL‐5Tg mice were originally generated by Lindsay A. Dent. A 10‐kb genomic mouse Il5 sequence under the control of the dominant control region (DCR) from human CD2 was used for the transgene. Δdbl GATA mice were originally generated by C. Yu. A high‐affinity, palindromic “double” GATA protein binding site in the Gata1 promoter presumed to mediate positive Gata1 autoregulation was replaced by a floxed Pgk‐neo cassette; transient Cre recombinase expression in ES cells left a single loxP site flanked by two Not1 sites. The 21‐bp deleted segment comprised nucleotides −691 through −671 upstream of the last nucleotide of the first haematopoietically expressed exon of Gata1. Mice were housed in plastic sawdust floor cages at constant temperature (22°C) and a 12:12‐h light–dark cycle with free access to standard laboratory chow and water; 8‐ to 12‐week‐old male and female mice were included in experiments, where there were randomly divided in three groups (negative control—vehicle; positive control—ovalbumin or eotaxin stimulated and miltefosine pretreated and ovalbumin or eotaxin stimulated group). Experiments, where bronchoalveolar lavage fluid was collected, could not be performed blinded, due to investigator treating the mice prior to fluid collection. Lung function testing was performed blinded, since Investigator 1 treated the mice and Investigator 2 independently performed lung function testing on mice in a random order. For all animal experiments, at least five mice were included in each group and at least two repeat experiments were carried out. Experiments were designed to make sample sizes relatively equal and randomized among comparison groups. Sample sizes were determined according to previous studies with similar analyses (Knuplez, Curcic, et al., 2020; Theiler et al., 2019).

### 2.3 Blood sampling and eosinophil isolation

Blood sampling from healthy volunteers was approved by the Institutional Review Board of the Medical University of Graz (17‐291 ex 05/06). All participants signed a written informed consent.

Polymorphonuclear leukocytes preparations were purified from citrated whole blood as previously described (Curcic et al., 2015). Firstly, platelet‐rich plasma was removed by centrifugation. Next, red blood cells and platelets were removed by dextran sedimentation and polymorphonuclear leukocytes preparations were obtained by density gradient separation. Eosinophils were isolated from polymorphonuclear leukocytes by negative magnetic selection using a cocktail of biotin‐conjugated antibodies against CD2, CD14, CD16, CD19, CD56 (neural cell adhesion molecule 1), CD123 (interleukin 3 receptor, α subunit) and CD235a (glycophorin A) as well as Anti‐Biotin MicroBeads from Miltenyi Biotec (Bergisch Gladbach, Germany). Eosinophil purity was determined by morphological analysis of Kimura‐stained cells and was typically greater than 97%.

### 2.4 Eosinophil shape change

Eosinophil shape change was determined as described previously (Luschnig‐Schratl et al., 2011). Approximately 5 × 10^4^ eosinophils per sample were suspended in assay buffer with Ca^2+^ and Mg^2+^, preincubated with miltefosine in different concentrations (15 min, room temperature [RT]) and then stimulated in water bath (4 min, 37°C) with eotaxin‐1 (CCL11). Afterwards, cells were transferred to ice and ice‐cold fixative solution was added to terminate the reaction and maintain the change in cell shape until analysis. The samples were analysed on a FACS Canto II flow cytometer (Becton Dickinson, Mountain View, CA, USA), where shape change was determined as the increase in the forward scatter (FSC) property of the cell and was normalized to unstimulated vehicle control.

### 2.5 CD11b (integrin, alpha M subunit (complement component 3 receptor 3 subunit) up‐regulation

CD11b up‐regulation on eosinophils was determined as described in detail elsewhere (Knuplez, Curcic, et al., 2020). Briefly, eosinophils were stained with anti‐CD11b–FITC, pretreated either with vehicle or miltefosine (20 μM) and stimulated with eotaxin‐2 (CCL24) as indicated in the figure legend. Additionally, CD11b up‐regulation assay was performed in the polymorphonuclear leukocytes fraction (see the Supporting Information), where cells were pre‐stained with anti‐CD16‐PE to distinguish between eosinophil and neutrophil polymorphonuclear leukocytes fractions. CD11b up‐regulation on neutrophils (CD16 + cells) was induced with *N*‐formylmethionyl‐leucyl‐phenylalanine (fMLP). Eosinophil or neutrophil CD11b expression was determined by flow cytometry as the geometric mean of the fluorescence in the FITC channel and expressed as per cent of unstimulated vehicle response.

### 2.6 CD63 expression

Eosinophils were stained with anti‐CD63‐FITC (1:100) and preincubated with vehicle or miltefosine (20 μM) (15 min, RT). Subsequently cells were primed with cytochalasin B (5 μg·ml^−1^) and stimulated with C5a in two different concentrations (Schratl et al., 2006). Degranulation was analysed by flow cytometry and expressed as fold increase in fluorescence over indicated vehicle mean.

### 2.7 Preparation of bone marrow‐derived eosinophils (BMDEs)

Mouse eosinophils were derived from bone marrow of BALB/c mice as described before (Dyer et al., 2008; Kienzl et al., 2020; Knuplez, Krier‐Burris, et al., 2020). Briefly, following the lysis of erythrocytes in bone marrow, the cells were cultured in RPMI + 20% HyClone FBS (GE Healthcare; # 10309433), 1% P/S, 25‐mM HEPES (Thermo Fisher; # 15630–080), 1× non‐essential amino acids (Thermo Fisher; # 11140–035), 1‐mM sodium pyruvate (Thermo Fisher; # 11360–039) and 50‐μM β‐mercaptoethanol (Sigma‐Aldrich; M3148) supplemented with 100‐ng·ml^−1^ stem cell factor (PreproTech; # 250–03) and 100‐ng·ml^−1^ FLT3L (PreproTech; # 250–31 L). On Day 4, medium was changed to media supplemented with 10‐ng·ml^−1^ IL‐5 (Bio‐Techne; # 405‐Ml‐005) only, to differentiate progenitors into eosinophils. On Day 14, purity and viability of bone marrow‐derived eosinophils (BMDEs) was assessed by flow cytometry staining for mouse eosinophil markers CCR3 (CCR3‐BV421) and Siglec‐F (Siglec‐F‐PE), and PI respectively. Cytospins of BMDEs were prepared, stained with a Hemacolor Rapid staining of blood smear and imaged on an Olympus BX41 microscope (Olympus, Vienna, Austria). Day 14 BMDEs were used for further *in vitro* analyses.

### 2.8 Calcium flux

Isolated human or differentiated mouse eosinophils were loaded with 2 μM of Fluo‐3 AM in the presence of 0.02% pluronic F‐127 for 1 h at RT in the dark. Individual samples were treated as indicated in the figure legend. Changes in [Ca^2+^]_I_ were detected as fluorescence in the FL1 channel by a FACSCalibur flow cytometer (Becton Dickinson, Mountain View, CA, USA), as described previously (Heinemann et al., 2003; Knuplez, Curcic, et al., 2020).

### 2.9 *In vitro* chemotaxis

Purified eosinophils were pretreated with either vehicle or miltefosine in different concentrations (15 min, RT) and were allowed to migrate to 10‐nM eotaxin‐2 (CCL24) in an HTS Transwell 96‐well plate with a 5‐μm pore size polycarbonate membrane (1 h, 37°C). Eosinophils that have migrated to the lower compartment were enumerated for 1 min by flow cytometric counting on a FACS Canto II (Becton Dickinson, Mountain View, CA, USA) (Knuplez, Curcic, et al., 2020).

### 2.10 Flow cytometric analysis of intracellular kinase phosphorylation

Isolated eosinophils were pretreated with either vehicle or miltefosine 20 (μM) (15 min, RT). Following the pretreatment, cells were incubated with 10‐nM eotaxin‐1 (CCL11) (3 min, 37°C). Subsequently, cells were fixed, permeabilized and stained as described previously (Knuplez, Curcic, et al., 2020). Phosphorylation of Akt residues in fixed eosinophils was quantified as the increase of fluorescence in the FITC fluorescence channel from unstimulated control.

### 2.11 Apoptosis

Eosinophil survival after preincubation with vehicle, positive control formaldehyde (3.8%) or miltefosine (5–40 μM) for different time points at 37°C was assessed by annexin V/PI staining, as described previously (Heinemann et al., 2005; Knuplez, Curcic, et al., 2020).

### 2.12 *In vivo* chemotaxis

In vivo eosinophil migration was induced by intranasal application of 4‐μg eotaxin‐2 CCL24 in 8‐week‐old male and female heterozygous IL‐5 transgenic (IL‐5Tg) mice (BALB/c background). The mice and their littermate controls received oral gavages of miltefosine (20 mg·kg^−1^ in 0.9% NaCl) or vehicle for three consecutive days before CCL24 application. Bronchoalveolar lavage fluid was collected 4 h after experiment had started. Migration of eosinophils was evaluated by flow cytometric counting of highly granular (high side scatter) CD11c^−^/Siglec‐F^+^ cells, as described previously (Knuplez, Curcic, et al., 2020). The gating strategy for evaluation of other immune cells was previously published (Knuplez, Curcic, et al., 2020) and was as follows: alveolar macrophages (Siglec‐F^+^/CD11c^+^), neutrophils (Siglec‐F^−^/CD11b^+^/Ly6G^+^), B cells (CD11c^−^/CD11b^−^/MHCII^+^), T cells (CD11c^−^/CD11b^−^/CD3^+^), dendritic cells (Siglec‐F^−^/Ly6G^−^/Ly6C^−^/MHCII^+^) and inflammatory monocytes (Siglec‐F^−^/Ly6G^−^/MHCII^−^/LY6C^++^).

### 2.13 Mouse model of allergic lung inflammation

Eight‐week‐old male and female BALB/c or eosinophil‐deficient (Δdbl GATA‐1) mice were immunized by intraperitoneal injections of 20 μg of ovalbumin adsorbed to Al (OH)_3_ on Days 0 and 7. Mice were challenged by an aerosol of ovalbumin (1 mg·ml^−1^ in 0.9% NaCl) on Days 14 and 16. During the last 10 days of the model, mice received daily oral gavages of miltefosine (20 mg·kg^−1^ in 0.9% NaCl) or vehicle. On Day 17, either airway hyperresponsiveness to methacholine was recorded with the FlexiVent system (SCIREQ, Montreal, QC, Canada) or bronchoalveolar lavage fluid was taken and analysed by flow cytometry. Bronchoalveolar lavage fluid supernatants were collected and stored at −70°C for further cytokine assessment. All animal subjects were randomized prior to inclusion in the experiments.

### 2.14 Cytokine measurements in bronchoalveolar lavage fluid

Cytokine concentrations in stored bronchoalveolar lavage fluid supernatants from BALB/c and Δdbl GATA‐1 mice subjected to ovalbumin/aluminium hydroxide were evaluated using the custom ProcartaPlex™ immunoassay (eBioscience, San Diego, CA, USA) according to the manufacturer's specifications. Fluorescent signals were quantified with the Bio‐Plex 200 multiplex suspension array system equipped with Luminex® xMAP® technology combined with the Bio‐Plex 5.0 software (Bio‐Rad, Hercules, CA, USA). All cytokine concentrations were evaluated in duplicates.

### 2.15 Corticosterone measurement in plasma

Corticosterone levels were assessed in plasma of BALB/c mice treated with oral gavages of miltefosine (20 mg·kg^−1^) once daily for 3 days. A blood sample was collected via cheek bleed 5 h after first miltefosine application on Day 1, as well as 4 h after last treatment on Day 3. Corticosterone levels were determined with a specific enzyme immunoassay kit (Assay Designs, Ann Arbor, MI, USA) with a sensitivity of 0.027 ng·ml^−1^ as previously described (Farzi et al., 2015) and according to the manufacturer's specifications.. The Immuno‐related procedures used comply with the recommendations made by the *British Journal of Pharmacology* (Alexander et al., 2018).

### 2.16 Statistical analysis

The data and statistical analysis comply with the recommendations of the *British Journal of Pharmacology* on experimental design and analysis in pharmacology (Curtis et al., 2018). Statistical analysis was performed using the GraphPad Prism™ 6 software (GraphPad Software, Inc., CA, USA). Data were normalized to baseline (1 or 100%) of the means of negative control in experiments performed with eosinophils isolated from human donors to reduce interindividual source of variation.

Statistical analysis was only performed for groups where *n* ≥ 5. Additional preliminary data (*n* = 3) on p‐Akt phosphorylation in eosinophils were included in the manuscript to suggest a mechanism previously shown for other cell types (Chugh et al., 2008; Ruiter et al., 2003). The group size given for each experiment is the number of independent values (individual human eosinophil donors or mice). Statistical analysis was performed using these independent values.

Data were tested for normality using D'Agostino and Pearson omnibus normality test. If normality was assumed, comparisons among multiple groups were performed with one‐way ANOVA or two‐way ANOVA. For these analyses, post hoc pairwise comparisons were performed using Bonferroni's multiple comparison test (or Dunnett's multiple comparison test, when comparing samples to the control group), only if a main effect for at least one factor or the interaction between two factors showed statistical significance and if there was no significant variance in homogeneity. Cytokine levels were compared using Mann–Whitney *U* test. Significance level for the analyses was set to *α* = 0.05 and significant differences are indicated with the corresponding *P* value, *P ≤ 0.05.

### 2.17 Nomenclature of targets and ligands

Key protein targets and ligands in this article are hyperlinked to corresponding entries in the IUPHAR/BPS Guide to PHARMACOLOGY http://www.guidetopharmacology.org and are permanently archived in the Concise Guide to PHARMACOLOGY 2019/20 (Alexander et al., 2019).

## 3 RESULTS

### 3.1 Miltefosine suppresses eosinophil activation *in vitro*

First, we tested the viability of eosinophils after pretreatment with different concentrations of miltefosine. Importantly, miltefosine (up to 20 μM, in the presence of 1‐mg·ml^−1^ bovine serum albumin) showed no toxic effects on eosinophils (Figure S1).

**FIGURE 1:**
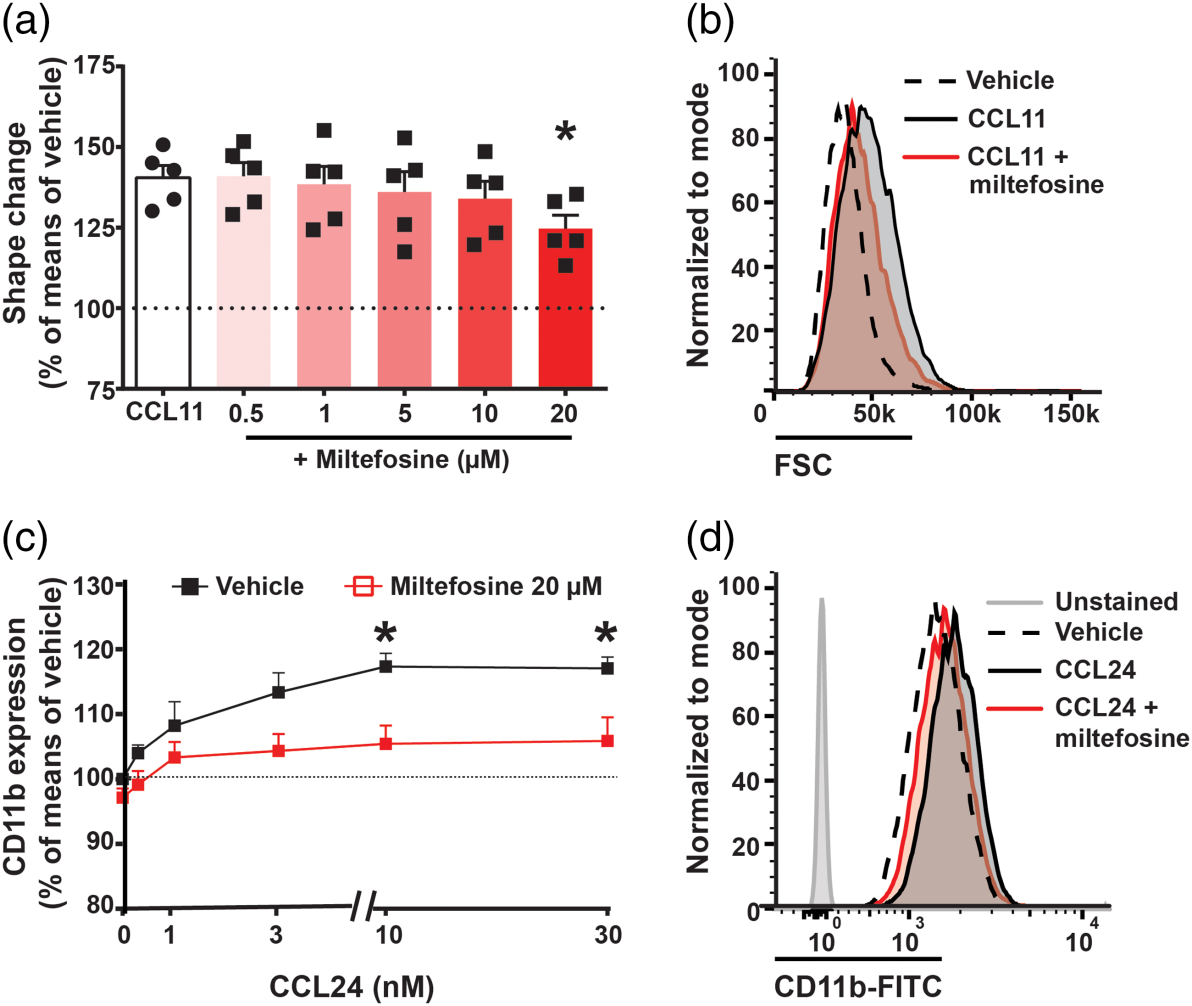
Miltefosine concentration‐dependently inhibits eosinophil activation. (a, b) Eosinophils were pretreated with miltefosine in different concentrations (0.5–20 μM) (15 min, room temperature [RT]) and stimulated with 10‐nM CCL11 (4 min, 37°C). Cells were fixed and the change in cell size (forward scatter [FSC]) was evaluated by flow cytometry. (a) Eosinophil shape change is expressed as per cent of unstimulated vehicle response. Data are shown as mean + SEM from five individual experiments. *P < 0.05 versus CCL11‐stimulated vehicle (one‐way ANOVA with Dunnett's post hoc test). (b) Representative histogram of eosinophil FSC with miltefosine (20 μM) pretreatment and CCL11 stimulation (10 nM). (c, d) Eosinophils were stained with anti‐CD11b and treated with either miltefosine (20 μM) or vehicle control (15 min, RT). Subsequently, cells were stimulated with CCL24 (4 min, 37°C) and analysed by flow cytometry. Eosinophil CD11b expression is expressed as per cent of unstimulated vehicle response. Data are shown as mean + SEM from five individual experiments. *P < 0.05 versus vehicle (two‐way ANOVA with Bonferroni post hoc test). (d) Representative histogram of CD11b up‐regulation with miltefosine (20 μM) pretreatment and CCL24 (10 nM) stimulation

During the state of allergic inflammation, elevated concentrations of cytokines and chemoattractants in the blood activate eosinophils, which leads to a rearrangement of their actin filaments (the so‐called “shape change”) (Willetts et al., 2014) and results in an up‐regulation of the adhesion molecules integrins (e.g., CD11b/CD18 and Mac‐1) on the cell surface (Jia et al., 1999). When human eosinophils were pretreated with miltefosine, we could observe a statistically significant inhibition of their shape change (by approx. 50%) induced by CCL11 stimulation (Figure 1a,b) when using the highest concentration of miltefosine (20 μM). Miltefosine addition did not alter eosinophil shape change in the absence of external stimuli (Figure S2). When isolated eosinophils were pretreated with 20‐μM miltefosine, up‐regulation of CD11b was reduced by about 50% (Figure 1c,d). Similarly, miltefosine suppressed CD11b expression of CCL11 activated eosinophils (CD16^−^ cells) in the polymorphonuclear leukocytes fraction (Figure S3A). Notably, miltefosine did not alter CD11b expression of fMLP‐stimulated neutrophils (CD16^+^ cells) (Figure S3B).

**FIGURE 2:**
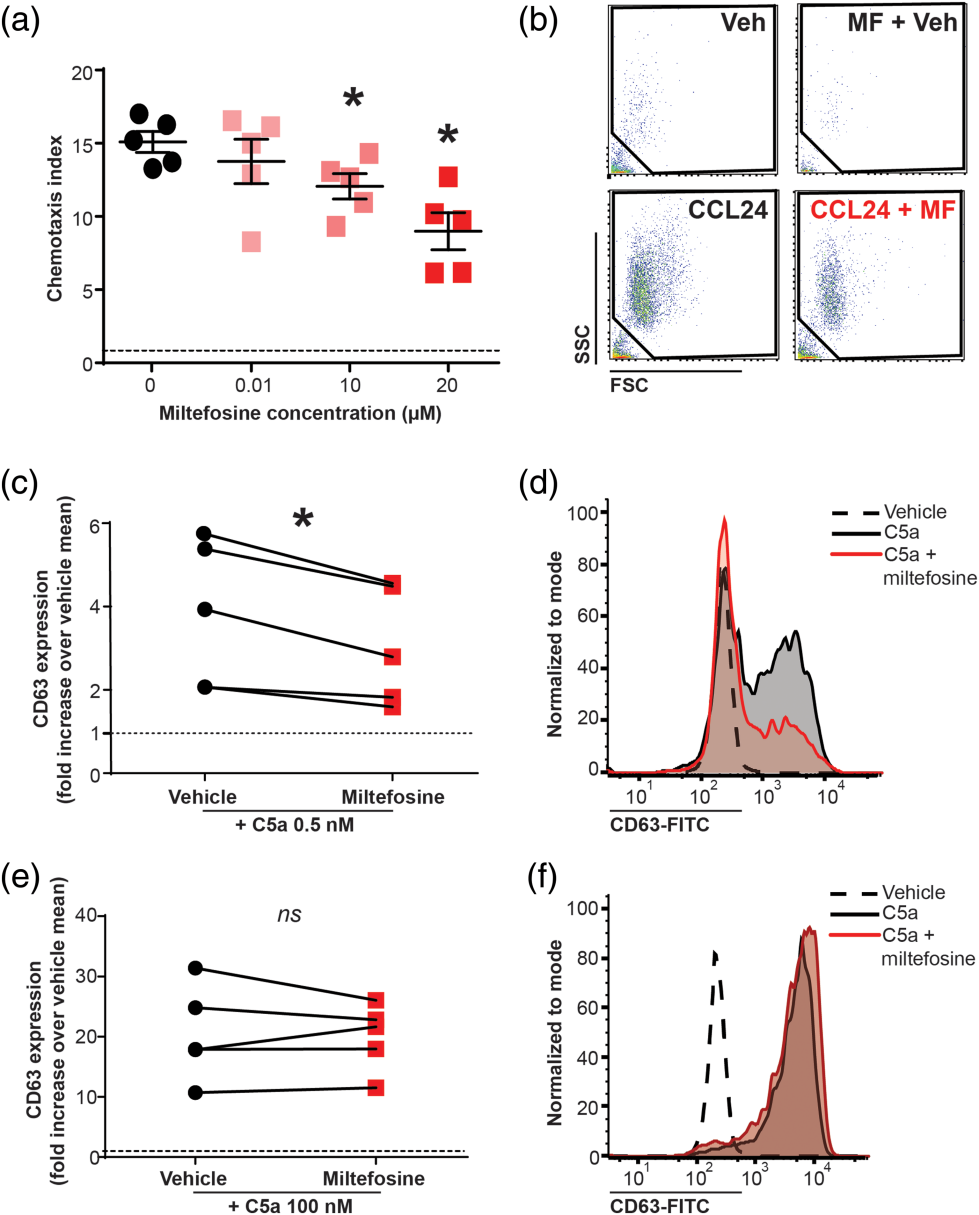
Miltefosine inhibits chemotaxis and CD63 expression of eosinophils. (a, b) Purified human eosinophils were treated with miltefosine in different concentrations (15 min, room temperature [RT]) or vehicle and allowed to migrate towards CCL24 (10 nM) in transwell plates (a). Migrated cells were enumerated by flow cytometry for 1 min. Results are presented as chemotactic index (migrated cell number/cell number migrated to unstimulated vehicle) and shown as mean ± SEM from five individual experiments. *P < 0.05 versus vehicle (one‐way ANOVA with Dunnett's post hoc test). (b) Representative scatter blots of cells migrating towards vehicle/miltefosine (20 μM) in the bottom compartment or cells pretreated with vehicle or miltefosine (20 μM) migrating towards CCL24. (c–f) Eosinophils were stained with anti‐CD63‐FITC followed by incubation with miltefosine (20 μM) or vehicle (15 min, RT). Subsequently, cells were primed with cytochalasin B and stimulated with complement component 5a (C5a) 0.5 nM (c, d) or 100 nM (e, f) for 20 min at 37°C. CD63 expression is expressed as fold increase of fluorescence intensity over unstimulated vehicle for individual donors. *P < 0.05, miltefosine versus vehicle treated (paired Student's *t* test). CCL24 + MF, CCL24 + miltefosine; MF + Veh, miltefosine + vehicle; Veh, vehicle

To determine whether miltefosine has an effect on the chemotaxis of human eosinophils, we performed *in vitro* chemotaxis assays using eotaxin‐2 (CCL24) as chemoattractant. Miltefosine significantly inhibited eosinophilic chemotaxis in a dose‐dependent manner (Figure 2a,b).

We next assessed whether miltefosine affects degranulation‐associated processes in eosinophils. For that purpose, eosinophils were pretreated with miltefosine and subsequently stimulated with recombinant C5a, a potent inducer of degranulation. Miltefosine effectively suppressed C5a (0.5 nM)‐induced CD63 expression, a marker of eosinophilic degranulation (Carmo et al., 2016) (Figure 2c,d). CD63 expression induced with very high concentrations of C5a (100 nM) was not affected by miltefosine (Figure 2e,f).

**FIGURE 3:**
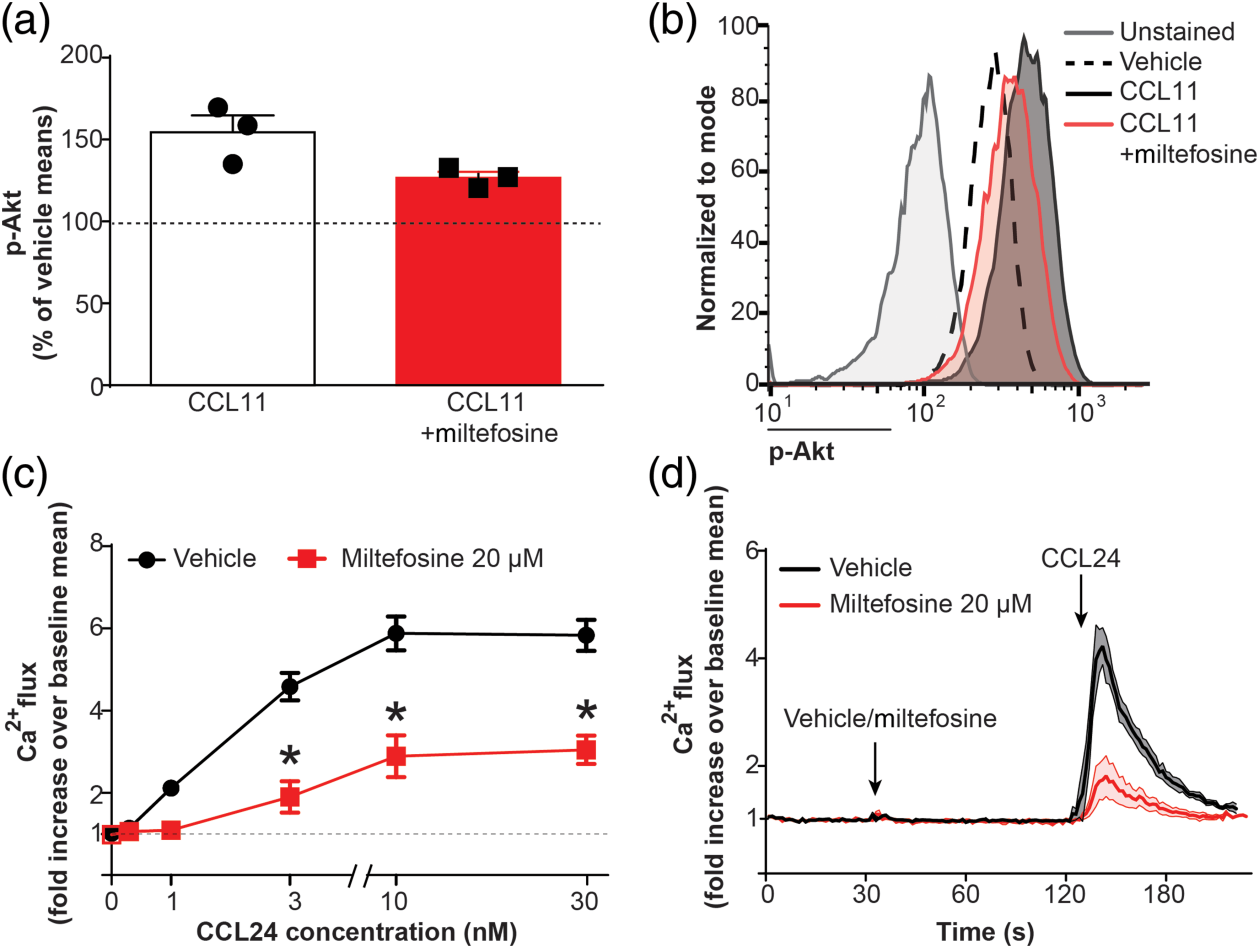
Miltefosine inhibits Akt phosphorylation and Ca^2+^ flux in human eosinophils. (a, b) Eosinophils were pretreated with miltefosine (20 μM) or vehicle (15 min, room temperature [RT]) followed by addition of CCL11 (3 min, 37°C). Subsequently, cells were fixed, permeabilized and stained. (a) Phosphorylation of Akt residues was quantified as the increase of fluorescence in the FITC fluorescence channel and expressed as per cent of unstimulated vehicle control. Data are shown as mean + SEM from three individual experiments. (b) Representative histogram of p‐Akt staining following miltefosine pretreatment. (c, d) Eosinophils were labelled with Fluo‐3 AM and changes in [Ca^2+^]_I_ were detected by flow cytometry. Eosinophils were stimulated with increasing concentrations of CCL24 (0–30 nM) in the presence or absence of miltefosine (20 μM). (c) Results represent fold increase in [Ca^2+^]_I_ over unstimulated vehicle. Data are shown as mean ± SEM from five individual experiments *P < 0.05 miltefosine (20 μM) versus vehicle (two‐way ANOVA with Bonferroni post hoc test). (d) Time course of Ca^2+^ flux in eosinophils. Following baseline measurement (30 s), miltefosine (20 μM) or vehicle was added. After 1 min, CCL24 (10 nM) was added to induce Ca^2+^ flux. Data are shown as mean ± SEM from five individual experiments

Previous studies have shown that miltefosine inhibits PI3K/Akt kinase signalling with an IC_50_ in the range of 5 to 35 μM, depending on the cell line tested (Kaleağasıoğlu et al., 2018; Rybczynska et al., 2001). Our preliminary results show a tendency of miltefosine (20 μM) inhibiting Akt phosphorylation (Figure 3a,b). Moreover, we could demonstrate that intracellular calcium flux in CCL11‐stimulated eosinophils was inhibited by about 50% (Figure 3c,d) after only 1 min of miltefosine addition (20 μM).

### 3.2 Miltefosine ameliorates ovalbumin‐induced lung inflammation

**FIGURE 4:**
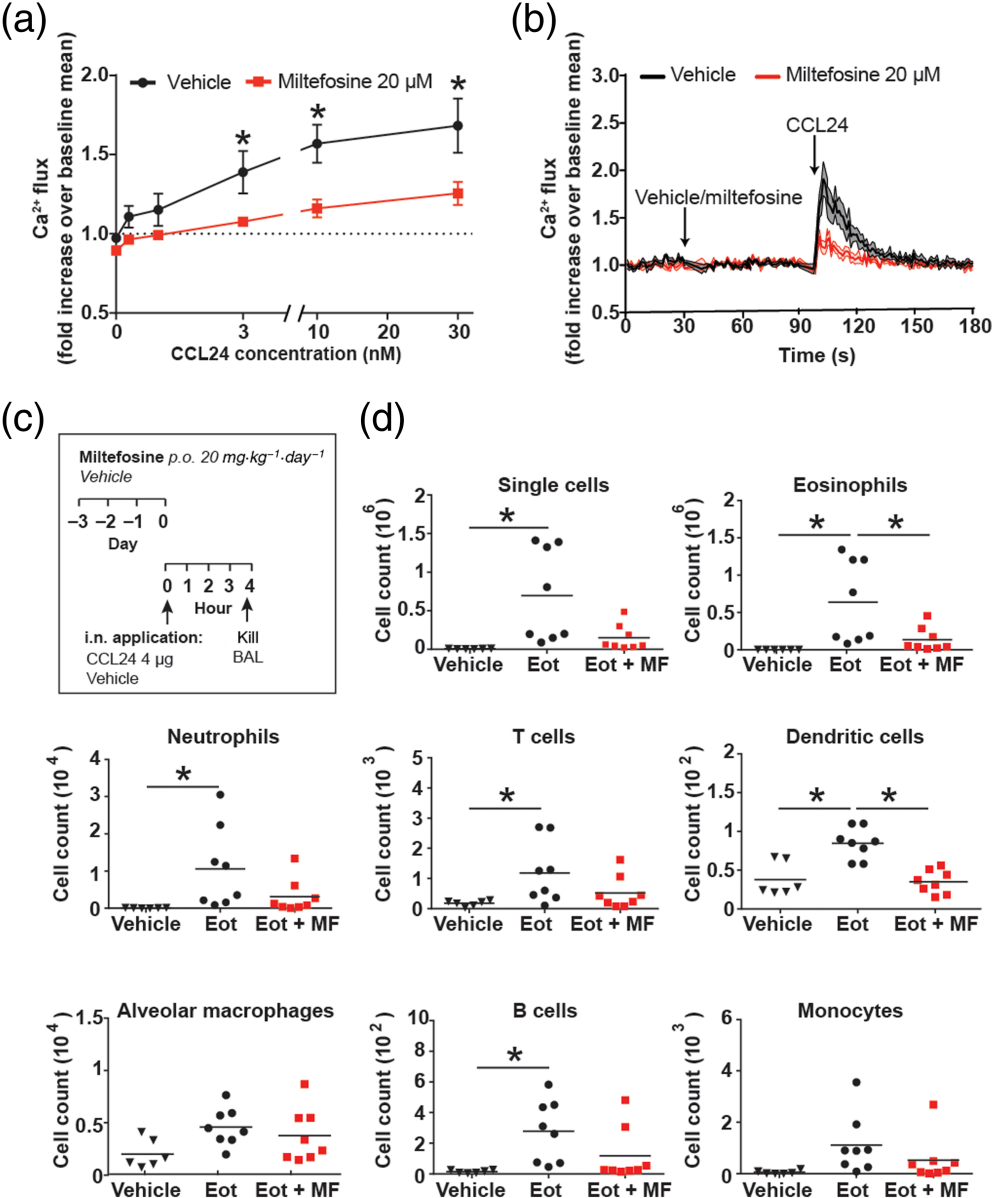
Miltefosine inhibits Ca^2+^ flux in mouse eosinophils and suppresses migration of eosinophils in vivo. (a, b) Bone marrow‐derived mouse eosinophils were labelled with Fluo‐3 AM and changes in [Ca^2+^]_I_ were detected by flow cytometry. Eosinophils were stimulated with increasing concentrations of CCL24 (0–30 nM) in the presence or absence of miltefosine (20 μM). (a) Results represent fold increase in [Ca^2+^]_I_ over unstimulated vehicle. Data are shown as mean ± SEM from five individual experiments *P < 0.05 miltefosine (20 μM) versus vehicle (two‐way ANOVA with Bonferroni post hoc test). (b) Time course of Ca^2+^ flux in eosinophils. Following baseline measurement (30 s), miltefosine (20 μM) or vehicle was added. After 1 min, CCL24 (10 nM) was added to induce Ca^2+^ flux. Data are shown as mean ± SEM from five individual experiments. (c, d) Eight‐week‐old IL‐5Tg mice received either miltefosine (20 mg·kg^−1^) or vehicle (0.9% NaCl) per os for three consecutive days, followed by intranasal application of CCL24 (4 μg) or vehicle. After 4 h, mice were killed and bronchoalveolar lavage (BAL) fluid was collected. (b) Immune cell composition in BAL fluid was analysed by flow cytometry. Data are shown from six to eight mice from three individual experiments. *P < 0.05 versus eotaxin (CCL24) (Eot) group (one‐way ANOVA with Dunnett's post hoc test). Eot + MF, eotaxin (CCL24) + miltefosine

Next, we investigated whether the *in vitro* results obtained with isolated human eosinophils are also relevant *in vivo*. We first performed Ca^2+^ flux assays using mouse bone marrow‐derived eosinophils to test whether mouse eosinophils behave similar to human‐isolated eosinophils (Figure 4a,b). For that purpose, eosinophils were differentiated from bone marrow cells of BALB/c mice following an established protocol (Kienzl et al., 2020), which yields a pure population of cultured eosinophils as determined by a single population positive for mouse eosinophil markers CCR3 and Siglec‐F (Figure S4A). Microscopic analysis of cytospins of BMDEs shows a uniform population of cells exhibiting typical eosinophil staining and granule morphology (Figure S4B). These mature bone marrow eosinophils were used to perform Ca^2+^ flux assays under similar experimental conditions as isolated human eosinophils (Figure 4a,b). Our data show the level (approximately 50%) and kinetic of Ca^2+^ flux inhibition in mouse eosinophils resembling that of human eosinophils pretreated with miltefosine and stimulated with CCL24 (Figure 4a,b).

Next, we performed an *in vivo* eosinophilic migration test using IL‐5Tg mice. This strain of mice is characterized by eosinophilia due to increased production of IL‐5. Together, intranasal eotaxin application in IL‐5‐primed eosinophils results in abundant and eosinophil accumulation in the bronchoalveolar lavage fluid and lungs of animals (Ochkur et al., 2007). We treated IL‐5Tg mice for three consecutive days perorally with miltefosine (20 mg·kg^−1^) (Figure 4c). We used a dosing regimen comparable with other studies in mice testing miltefosine (Bäumer et al., 2010; Dorlo et al., 2012). Remarkably, miltefosine significantly suppressed the migration of eosinophils to intranasal CCL24 into the bronchoalveolar lavage of animals (Figure 4d). A trend towards reduced infiltration of immune cells was observed for all detected cell types (Figure 4d). Analysis of the blood immune cell composition revealed an increase in the percentage of neutrophils in blood of IL‐5Tg mice treated with miltefosine (Figure S5A); however, when BALB/c mice were treated with miltefosine, no increase in neutrophils was observed (Figure S5B). By testing plasma of BALB/c mice for their corticosterone levels, we observed no significant differences at both of the two tested time points (Figure S6A,B).

**FIGURE 5:**
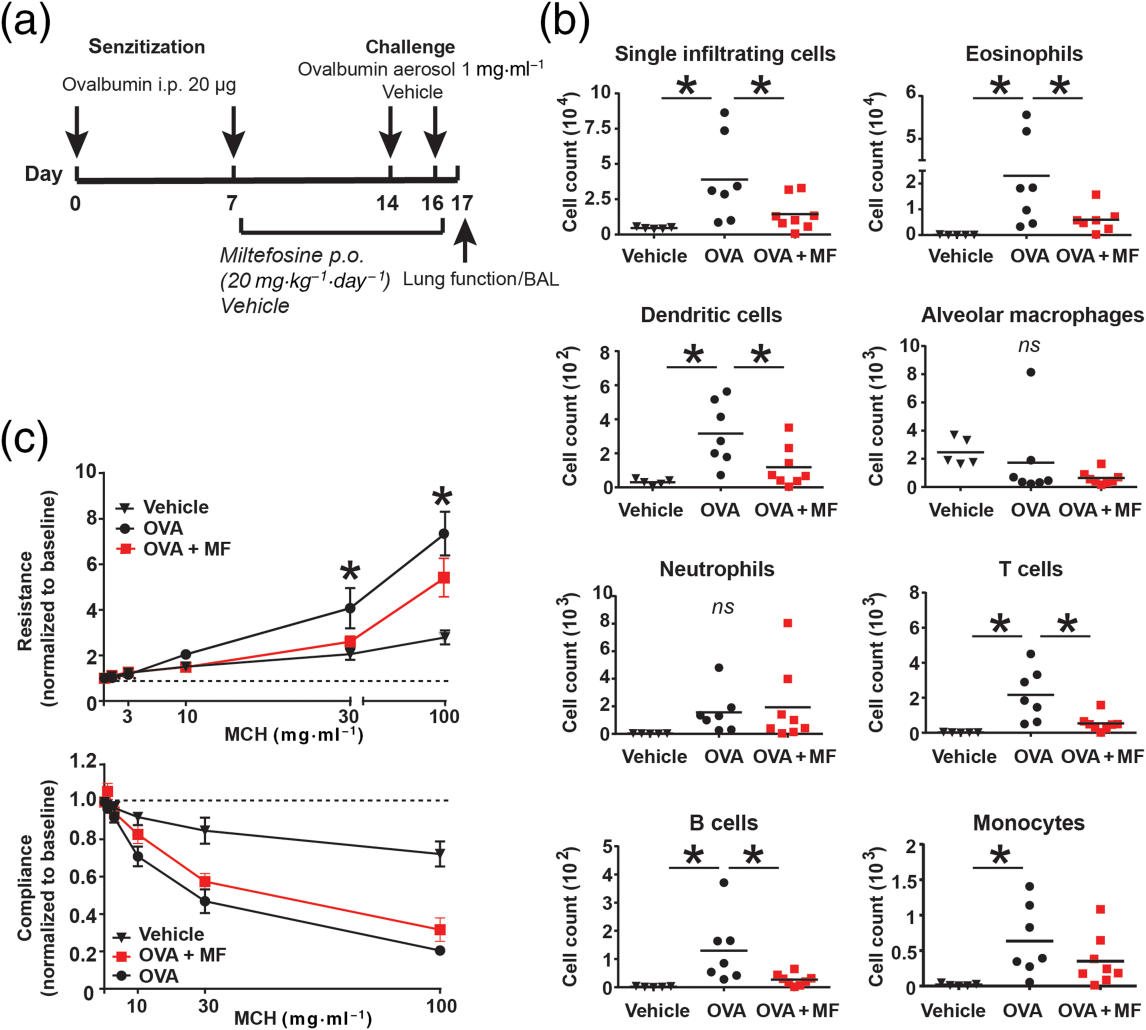
Miltefosine ameliorates ovalbumin‐induced allergic lung inflammation. (a) Eight‐week‐old BALB/c mice were sensitized with ovalbumin intraperitoneal at Day 0 and at Day 7 and were subsequently treated with miltefosine (20 mg·kg^−1^) or vehicle per os daily from Day 7 to Day 17. Afterwards, mice were challenged with an ovalbumin aerosol on Day 14 and Day 16 followed by lung function testing or sampling of bronchoalveolar lavage (BAL) fluid. (b) Immune cell composition in the BAL fluid was analysed by flow cytometry. Representative results from two individual experiments are shown. *P < 0.05 versus OVA group (one‐way ANOVA with Dunnett's post hoc test). (c) Lung function of mice was assessed while applying increasing doses of methacholine (0–100 mg·ml^−1^). Data are shown as mean ± SEM from two individual experiments performed with 5–8 mice. *P < 0.05, ovalbumin (OVA) + miltefosine (MF) versus OVA group (two‐way ANOVA with Dunnett's post hoc test). MCH, methacholine

**FIGURE 6:**
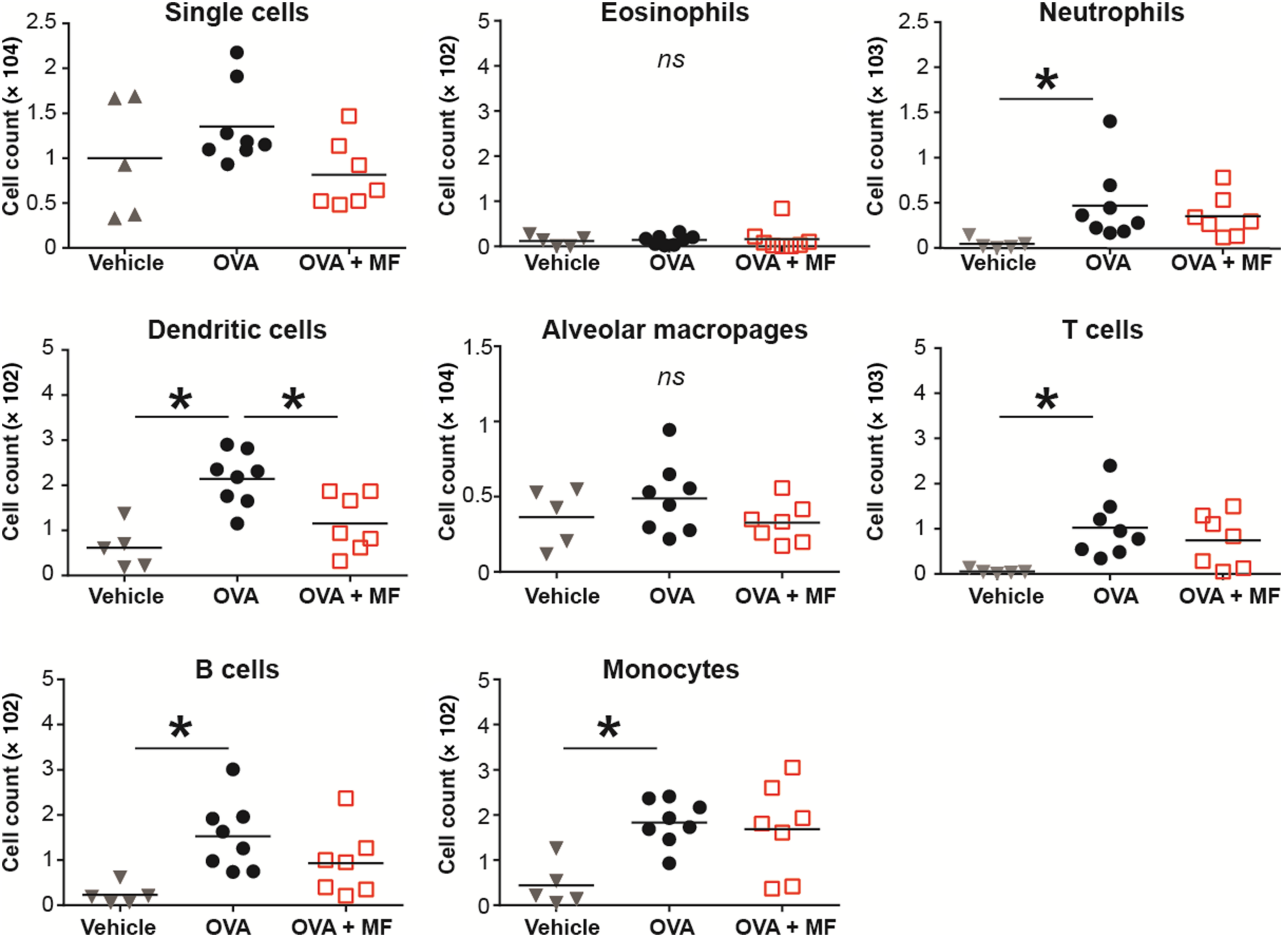
The effect of miltefosine treatment in eosinophil‐deficient mice. Eight‐week‐old Δdbl GATA‐1 mice were sensitized with ovalbumin intraperitoneal at Day 0 and at Day 7 and subsequently treated with miltefosine (20 mg·kg^−1^) or vehicle per os daily from Day 7 to Day 17. Afterwards, mice were challenged with an ovalbumin aerosol on Day 14 and Day 16 followed by sampling of bronchoalveolar lavage (BAL) fluid. Immune cell composition in the BAL fluid was analysed by flow cytometry. Results from two individual experiments with 5–7 mice are shown. *P < 0.05 versus ovalbumin (OVA) group (one‐way ANOVA with Dunnett's post hoc test). OVA + MF, ovalbumin + miltefosine

We next tested the efficacy of miltefosine in an acute model of allergic lung inflammation. Ovalbumin was used as a model allergen to reproduce key features of clinical asthma, such as airway hyperresponsiveness to methacholine (Kumar et al., 2008). The treatment protocol of the model is shown in Figure 5a. We observed that daily peroral treatment with miltefosine markedly reduced the number of several infiltrating immune cells into airways of ovalbumin‐challenged wild‐type mice. Flow cytometric analysis of the composition of immune cells showed that the number of eosinophils as well as infiltrating T cells, B cells and dendritic cells was reduced by 50% upon miltefosine treatment (Figure 5b). Of note, mice treated with miltefosine showed significantly improved lung resistance and a trend towards improved lung compliance (Figure 5c). In order to test whether a decrease in eosinophil numbers was responsible for the reduction of other immune cells, eosinophil‐deficient (Δdbl GATA‐1) mice were exposed to the same ovalbumin‐induced allergic model. In this mouse strain, treatment with miltefosine only had an impact on the number of dendritic cells (Figure 6), while other subgroups of immune cells were not affected.

Supernatants of bronchoalveolar lavage fluid of ovalbumin stimulated (vehicle) and miltefosine‐treated and ovalbumin‐stimulated BALB/c and Δdbl GATA‐1 mice were further analysed for their cytokine content (Figures S7 and S8). We could observe significantly reduced levels of immunomodulatory cytokine IFN‐γ in the bronchoalveolar lavage fluid of miltefosine‐treated BALB/c mice (Figure S7A), while no such inhibition was observed in Δdbl GATA‐1 mice (Figure S8A). Cytokine content of CCL11, TNF‐α, IL‐2 and IL‐5 in bronchoalveolar lavage fluid was not significantly altered by miltefosine treatment in either BALB/c or Δdbl GATA‐1 mice (Figures S7 and S8).

## 4 DISCUSSION

In the present study, we show for the first time that the Food and Drug Administration (FDA)‐approved drug miltefosine inhibits the activation of human eosinophils. A short pretreatment with miltefosine suppressed human eosinophilic effector responses after stimulation with various agonists *in vitro*. We were able to transfer our *in vitro* findings to preclinically relevant endpoints in an *in vivo* model of eosinophilic migration and allergic inflammation. Most importantly, in a model of ovalbumin‐induced allergic lung inflammation, peroral administration of miltefosine significantly reduced the infiltration of immune cells into the lung while improving lung function parameters.

The effects of miltefosine have previously been studied on some other immune cells. Notably, miltefosine was found to inhibit degranulation and antigen‐induced chemotaxis of mast cells by modulating lipid rafts and by inhibiting cytosolic PKC (Rubíková et al., 2018). In contrast to our findings with eosinophils, calcium flux in mast cells was apparently not affected by miltefosine pretreatment, indicating cell type‐specific differences. However, similar to mast cells, miltefosine led to an inhibition of effector functions and mediator release in eosinophils. In macrophages, miltefosine was found to increase cholesterol release and phosphorylation of kinases associated with autophagy. Importantly, miltefosine decreased toll‐like receptor 4 (TLR‐4) recruitment to the cell surface of macrophages and dampened IL‐1β release following stimulation with lipopolysaccaride (LPS) (Iacano et al., 2019). Given the fact that TLR‐4 stimulation on eosinophils can help polarize macrophages towards pro‐ or anti‐inflammatory phenotypes (Yoon et al., 2019), this finding further supports the evidence that miltefosine may influence the interplay and balance between various immune cell types during the state of inflammation.

It is noteworthy that in all our *in vitro* experiments, non‐toxic concentrations of miltefosine were used to distinguish our results from the non‐specific cytolytic effects of the drug. In particular, since homeostatic functions such as tissue remodelling and plasma cell survival (Jacobsen et al., 2012) have recently been attributed to eosinophils, we were mainly interested in inhibiting eosinophil overactivation, to prevent their potential tissue‐damaging effector functions. For our *in vivo* experiments, we used a dosage regimen, comparable with other studies in mice testing miltefosine (Bäumer et al., 2010).

Results from our *in vivo* experiments show significantly decreased numbers of infiltrating eosinophils in miltefosine‐treated animals compared with vehicle‐challenged controls. The data correspond to our *in vitro* experiments in which miltefosine inhibited the activation, migration and up‐regulation of adhesion molecules on eosinophils. Interestingly, we additionally discovered a trend towards decreased numbers of other infiltrating immune cells in miltefosine‐treated animals, while the numbers of tissue resident alveolar macrophages remained the same across all treatment groups. In order to confirm whether infiltration of other immune cells is directly affected by miltefosine, we performed control experiments in eosinophil‐deficient Δdbl GATA‐1 mice. We discovered that the decreased infiltration of most immune cells was at least partially due to the decreased eosinophil numbers. This is not unexpected, since activated eosinophils are known to attract and activate other immune cell types such as neutrophils (Yousefi et al., 1995) or B cells (Chu et al., 2011). Moreover, eosinophil‐derived CCL17 and CCL22 have proven to be crucial in attracting effector T cells in localized allergic inflammation (Jacobsen et al., 2008). Interestingly, we observed a decrease in dendritic cell (CD11c^+^/MHCII^+^/Siglec‐F^−^/Ly6G^−^/Ly6C^−^) numbers following miltefosine treatment compared with challenged controls both in wild‐type and in eosinophil‐deficient mice. It has been reported previously that combination therapy of paromomomycin/miltefosine can influence TLR9 on dendritic cells and therefore modulate Th1 host immune responses in leishmaniasis therapy (Das et al., 2014). As of yet, however, the direct effect of miltefosine on human dendritic cells remains unclear.

To assess whether the observed differences in immune cell count are a consequence of miltefosine directly inhibiting immune cell infiltration or rather indirectly altering the cytokine milieu in the lung, we additionally tested bronchoalveolar lavage supernatants from both BALB/c and Δdbl GATA‐1 mice for cytokine expression. Cytokine concentrations of CCL11, TNF‐α, IL‐2 and IL‐5 were not altered in miltefosine‐treated and ovalbumin‐stimulated mice of both genotypes. Interestingly however, we observed significantly reduced levels of the immunomodulatory cytokine IFN‐γ in the miltefosine‐treated group of BALB/c mice. These data corroborate previous findings from Verhaar et al. (2013), where they observed similarly reduced levels of IFN‐γ in miltefosine‐treated animals in a mouse model of inflammatory bowel disease. IFN‐γ has on one hand long been considered to be beneficial in allergic inflammation as reviewed by Teixeira et al. (2005), while on the other hand, recent studies recognize its pro‐inflammatory functions. Our findings of reduced IFN‐γ expression in BALB/c mice and not in Δdbl GATA‐1 mice are of particular interest, since it was discovered that eosinophil‐derived IFN‐γ induces airway hyperresponsiveness and lung inflammation even in the absence of lymphocytes (Kanda et al., 2009). Interestingly, IFN‐γ was also found to up‐regulate several eosinophil effector functions (Ishihara et al., 1997; Takaku et al., 2011) and promote their survival (Fujisawa et al., 1994).

When we examined the composition of immune cells in mouse blood, miltefosine‐treated and CCL24‐stimulated IL‐5Tg animals showed an increased neutrophil count, yet miltefosine‐treated BALB/c animals showed no altered neutrophil numbers at baseline. A previous study showed that patients treated with miltefosine exhibited increased levels of the neutrophilic chemokine IL‐8 (CXCL8) (Mukhopadhyay et al., 2011. This finding remains to be confirmed in mice. Increased corticosterone levels in mice induced by miltefosine could be another plausible explanation for both increased neutrophil numbers (Liles et al., 1995) and decreased airway inflammation (Suqin et al., 2009). Furthermore, an inverse association between endogenous glucocorticoid and IFN‐γ levels was observed in allergic lung inflammation (Suqin et al., 2009). Nonetheless, we observed no significant alterations in corticosterone levels in miltefosine‐treated mice.

Fang et al. additionally showed that miltefosine acts on endothelial cells by down‐regulating E‐selectin, which is important for leukocyte adhesion and infiltration (Leung et al., 2007). Therefore, in our ovalbumin model of allergic inflammation, we cannot neglect additional anti‐inflammatory effects of miltefosine.In another point, our work raises important questions regarding the immunomodulatory effect of miltefosine in patients treated for leishmania infections. So far, little has been reported about the drug's effect on the host responses responsible for fighting the infection. However, some *in vitro* findings report a strong reversal of Th2 responses of leishmania‐infected macrophages towards Th1 type following miltefosine treatment (Wadhone et al., 2009). Since eosinophils are one of the primary cells recruited to the sites of leishmania infection (de Oliveira Cardoso et al., 2010) and have been shown to help control parasite load (Watanabe et al., 2004) in mice, it might be of interest to further investigate this issue in patients treated with miltefosine. In line with the present study, we have previously shown that saturated lysophosphatidylcholines, which are structurally similar to miltefosine, inhibit eosinophil effector responses (Knuplez, Curcic, et al., 2020; Knuplez, Krier‐Burris, et al., 2020; Trieb et al., 2019).

A limitation of our work needs to be noted. Ovalbumin was used as a model allergen in our in vivo studies, albeit this model fails to completely reflect the aetiology of human asthma and its multi‐step developmental process, including environmental factors associated with the disease. Further experiments with other physiological relevant antigens are needed to validate the relevance of our data in human disease setting.

In summary, we demonstrate the inhibitory effect of the orphan drug miltefosine on human eosinophils and its anti‐inflammatory effect *in vivo* in a model of allergic inflammation. Our data highlight the potential efficacy of miltefosine or related molecules in the treatment of allergic diseases and other eosinophil‐associated disorders.

## FUNDING INFORMATION

This study was supported by the Austrian Science Fund (FWF Grants W1241, DK‐MOLIN and P30144).

## AUTHOR CONTRIBUTIONS

E.K. designed and performed the experiments, analysed the data, interpreted the results and wrote the manuscript. A.T. and M.K. performed the experiments, analysed the data and edited the manuscript. E.M.S., A.H. and R.S. interpreted the results and edited the manuscript. G.M. designed and supervised the study, interpreted the results and wrote and edited the manuscript.

## CONFLICT OF INTEREST

A.H. received consultancy fees from AstraZeneca. The other authors declare no conflicts of interest.

## DECLARATION OF TRANSPARENCY AND SCIENTIFIC RIGOUR

This Declaration acknowledges that this paper adheres to the principles for transparent reporting and scientific rigour of preclinical research as stated in the *BJP* guidelines for Design & Analysis, Immunoblotting and Immunochemistry and Animal Experimentation, and as recommended by funding agencies, publishers and other organizations engaged with supporting research.

## Supporting information


**Figure S1.** Addition of albumin ameliorates apoptosis‐inducting effects of miltefosine. Eosinophils were pretreated with miltefosine or vehicle in indicated concentrations for either 30 min (A) or 2 h (B) at 37°C. Subsequently, cells were stained with Annexin V‐FITC or propidium iodide (PI). Results depict percent of viable cells (double negative) shown as mean + SEM from three individual experiments. *p < 0.05 vs vehicle (One‐Way ANOVA with Dunnett's post‐hoc test). (C) Eosinophils were pretreated with vehicle or miltefosine 50 μM (diluted in buffer or pre‐complexed to 1% solution of bovine serum albumin (BSA) in buffer) for 30 min at 37°C. Mean + SEM of double negative (viable) cells from two independent experiments is shown. *p < 0.05 vs miltefosine 50 μM (in buffer) (One‐Way ANOVA with Dunnett's post‐hoc test). (D) Representative scatter plots of Annexin V/Propidium iodide staining at time point 0 or after 30 min of pretreatment with vehicle, miltefosine (20 μM) or formaldehyde (positive control) are shown.
**Figure S2.** Miltefosine addition does not induce shape change of eosinophils. Eosinophils were stimulated with 10 nM CCL11 or miltefosine (0.5–20 μM) (4 min, 37°C). Cells were fixed and the change in cell size (FSC) was evaluated by flow cytometry. Eosinophil shape change is expressed as percent of unstimulated vehicle response.
**Figure S3.** Miltefosine does not inhibit CD11b upregulation on neutrophils. Polymorphonuclear leukocytes (PMNL) were stained with anti‐CD16 and anti‐CD11b and then treated either with miltefosine (20 μM) or vehicle control (15 min, RT). (A) Cells were stimulated with CCL24 for 4 min at 37°C and CD11b expression was analyzed by flow cytometry on CD16‐ cells (eosinophils) and expressed as percent of unstimulated vehicle control. (B) Cells were stimulated with fMLP for 4 min at 37°C and CD11b expression was analyzed by flow cytometry on CD16 + cells (neutrophils) and expressed as percent of unstimulated vehicle control. Data are shown as mean + SEM from five individual experiments. *p < 0.05, vs vehicle (Two‐Way ANOVA with Bonferroni post hoc test).
**Figure S4.** Differentiated bone marrow derived eosinophils (BMDEs) stain positive for mouse eosinophil markers and exhibit typical mouse eosinophil morphology. (A‐B) BMDEs were differentiated from bone marrow of BALB/c mice for 14 days. (A) Purity (Siglec‐F.positive, CCR3 positive) and viability (PI negative) was assessed with flow cytometry. Representative plot of eosinophils isolated from one mouse is shown. (B) Representative microscopy image of H&E stained cytospin of BMDEs from one mouse on day 14 of the differentiation protocol is shown, calibration bar: 20 μm.
**Figure S5.** Immune cell composition in blood. Eight week old IL‐5Tg (A) or BALB/c (B) mice received either miltefosine (20 mg/kg) or vehicle (0.9% NaCl) per os for three consecutive days. (A) On the last day mice received intranasal application of CCL24 or vehicle. Subsequently after four hours blood was collected and analyzed for immune cell composition. Data are shown as mean + SEM from 6‐8 mice from two individual experiments. *p < 0.05, vs eotaxin group (One‐Way ANOVA with Dunnett's post hoc test). (B) Four hours after last treatment blood was collected and analyzed for immune cell composition. Data are shown as mean + SEM from 10 mice from two individual experiments.
**Figure S6.** Corticosterone levels in plasma are not significantly altered following miltefosine treatment. (A‐B) BALB/c mice received either vehicle (0.9% NaCl) or miltefosine (20 mg/kg) per os for three consecutive days. (A) Blood was sampled five hours after first treatment on day 1 or four hours following last treatment on day 3 (B). Corticosterone levels were assessed with a specific enzyme immunoassay kit. Data are shown as mean ± SEM from 10 mice.
**Figure S7.** Miltefosine treatment decreases IFN‐γ in bronchoalveolar lavage (BAL) fluid of BALB/c mice in a model of allergic inflammation. Eight‐week‐old BALB/c mice were sensitized with ovalbumin intraperitoneal (i.p.) at day 0 and at day 7 and were subsequently treated with miltefosine (20 mg/kg) or vehicle per os daily from day 7 to day 17. Afterwards mice were challenged with an ovalbumin aerosol on day 14 and day 16 followed by sampling of BAL fluid. BAL fluid supernatants were subsequently analysed with a custom multiplex ELISA testing for IFN‐γ (A), CCL11 (B), IL‐5 (C), IL‐2 (D) and TNF‐α (E) levels. Data are shown as mean + SEM from 8 mice per group. Dotted line represents the lower limit of quantification (LLOQ) for each measured cytokine determined from its standard curve. *p < 0.05 analyzed with Mann‐Whitney U test.
**Figure S8.** Miltefosine treatment does not alter cytokine content in the bronchoalveolar lavage (BAL) fluid of eosinophil deficient mice. Eight‐week‐old Δdbl GATA‐1 mice were sensitized with ovalbumin intraperitoneal (i.p.) at day 0 and at day 7 and were subsequently treated with miltefosine (20 mg/kg) or vehicle per os daily from day 7 to day 17. Afterwards mice were challenged with an ovalbumin aerosol on day 14 and day 16 followed by sampling of BAL fluid. BAL fluid supernatants were subsequently analysed with a custom multiplex ELISA testing for IFN‐γ (A), CCL11 (B), IL‐5 (C), IL‐2 (D) and TNF‐α (E) levels. Data are shown as mean + SEM from 7 mice per group. Dotted line represents the lower limit of quantification (LLOQ) for each measured cytokine determined from its standard curve.

## Data Availability

The data that support the findings of this study are available from the corresponding author upon reasonable request. Some data may not be made available because of privacy or ethical restrictions.
