# Supplementary material for: The anti‐parasitic drug miltefosine suppresses activation of human eosinophils and ameliorates allergic inflammation in mice
Source: Br J Pharmacol. 2021 Feb 2;178(5):1234–48. doi: 10.1111/bph.15368 (PMC9328393; doi:10.1111/bph.15368)
Supplement: Supplementary file 1 — Figure S1. Addition of albumin ameliorates apoptosis‐inducting effects of miltefosine. Eosinophils were pretreated with miltefosine or vehicle in indicated concentrations for either 30 min (A) or 2 h (B) at 37°C. Subsequently, cells were stained with Annexin V‐FITC or propidium iodide (PI). Results depict percent of viable cells (double negative) shown as mean + SEM from three individual experiments. *p < 0.05 vs vehicle (One‐Way ANOVA with Dunnett's post‐hoc test). (C) Eosinophils were pretreated with vehicle or miltefosine 50 μM (diluted in buffer or pre‐complexed to 1% solution of bovine serum albumin (BSA) in buffer) for 30 min at 37°C. Mean + SEM of double negative (viable) cells from two independent experiments is shown. *p < 0.05 vs miltefosine 50 μM (in buffer) (One‐Way ANOVA with Dunnett's post‐hoc test). (D) Representative scatter plots of Annexin V/Propidium iodide staining at time point 0 or after 30 min of pretreatment with vehicle, miltefosine (20 μM) or formaldehyde (positive control) are shown. Figure S2. Miltefosine addition does not induce shape change of eosinophils. Eosinophils were stimulated with 10 nM CCL11 or miltefosine (0.5–20 μM) (4 min, 37°C). Cells were fixed and the change in cell size (FSC) was evaluated by flow cytometry. Eosinophil shape change is expressed as percent of unstimulated vehicle response. Figure S3. Miltefosine does not inhibit CD11b upregulation on neutrophils. Polymorphonuclear leukocytes (PMNL) were stained with anti‐CD16 and anti‐CD11b and then treated either with miltefosine (20 μM) or vehicle control (15 min, RT). (A) Cells were stimulated with CCL24 for 4 min at 37°C and CD11b expression was analyzed by flow cytometry on CD16‐ cells (eosinophils) and expressed as percent of unstimulated vehicle control. (B) Cells were stimulated with fMLP for 4 min at 37°C and CD11b expression was analyzed by flow cytometry on CD16 + cells (neutrophils) and expressed as percent of unstimulated vehicle control. Data are shown as me [file BPH-178-1234-s001.pdf]

Supplementary materials

The anti-parasitic drug miltefosine suppresses human eosinophil activation and ameliorates murine allergic inflammation in vivo

Knuplez E et al.,

Optimization of assay conditions

Prior to our assays, the viability of eosinophils after pretreatment with different miltefosine concentrations was assessed, with regards to the cytotoxic effects of miltefosine and structurally related compounds at higher concentrations (Ruiter et al., 2001; Dymond et al., 2008) (**Supplemental Figure S1 A-B**). The reported critical micellar concentration for miltefosine on model membranes in aqueous solution is 50 µM (Barioni et al., 2015). We observed toxic effects of miltefosine on eosinophils at similar concentrations. However, miltefosine toxicity (at higher concentrations) could be completely reversed by pre-complexing it with a 10 mg/ml solution of bovine serum albumin (BSA) (**Supplemental Figure S1 C**). In order to avoid non-specific toxic effects of miltefosine, all further *in vitro* assays were performed in concentrations up to 20 µM in assay buffer containing 1 mg/ml BSA (**Supplemental Figure S1 A-B, D**).

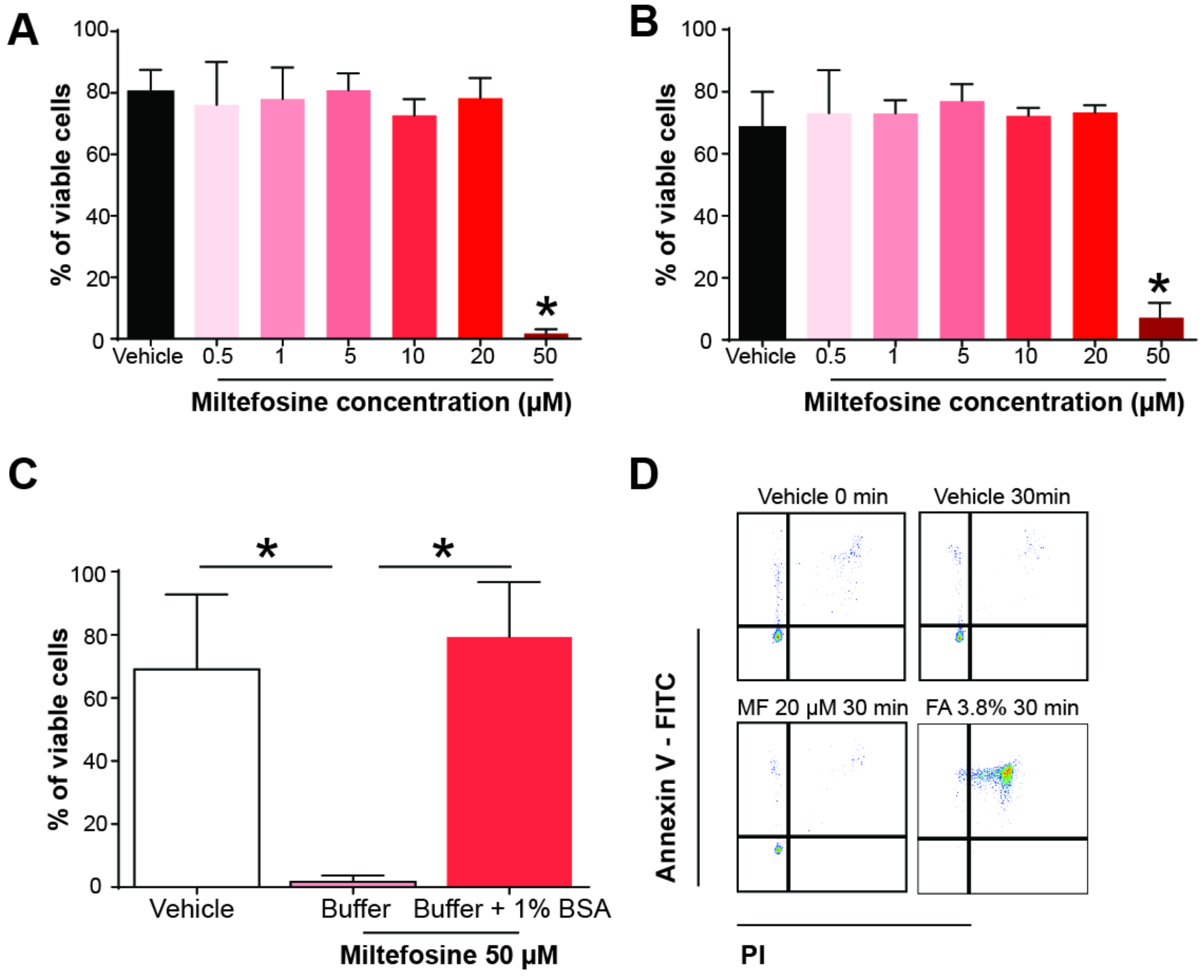

Figure S1. Addition of albumin ameliorates apoptosis-inducting effects of miltefosine. Eosinophils were pretreated with miltefosine or vehicle in indicated concentrations for either 30 min **(A)** or 2 h **(B)** at 37°C. Subsequently, cells were stained with Annexin V-FITC or propidium iodide (PI). Results depict percent of viable cells (double negative) shown as mean + SEM from three individual experiments. *p<0.05 vs vehicle (One-Way ANOVA with Dunnett´s post-hoc test). (**C**) Eosinophils were pretreated with vehicle or miltefosine 50µM (diluted in buffer or pre-complexed to 1% solution of BSA in buffer) for 30 min at 37°C. Mean + SEM of double negative (viable) cells from two independent experiments is shown. *p<0.05 vs miltefosine 50 µM (in buffer) (One-Way ANOVA with Dunnett´s post-hoc test). (**D**) Representative scatter plots of Annexin V/Propidium iodide staining at time point 0 or after 30 min of pretreatment with vehicle, miltefosine (20 µM) or formaldehyde (positive control) are shown.

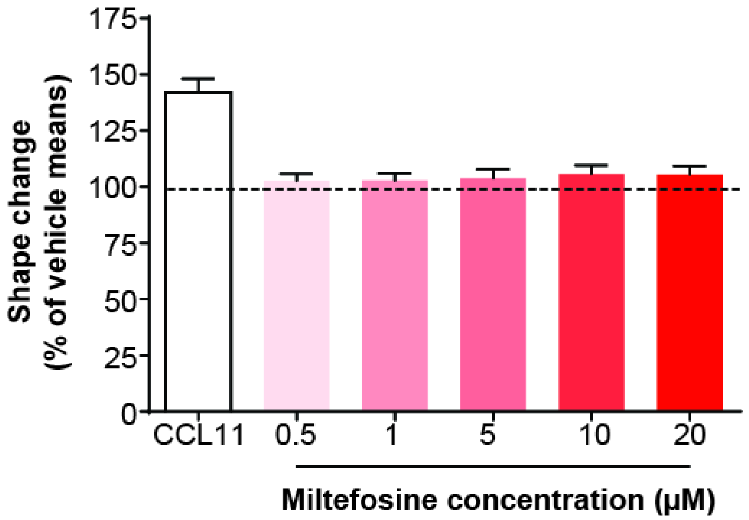

Figure S2. Miltefosine addition does not induce shape change of eosinophils. Eosinophils were stimulated with 10nM CCL11 or miltefosine (0.5–20 μM) (4min, 37°C). Cells were fixed and the change in cell size (FSC) was evaluated by flow cytometry. Eosinophil shape change is expressed as percent of unstimulated vehicle response.

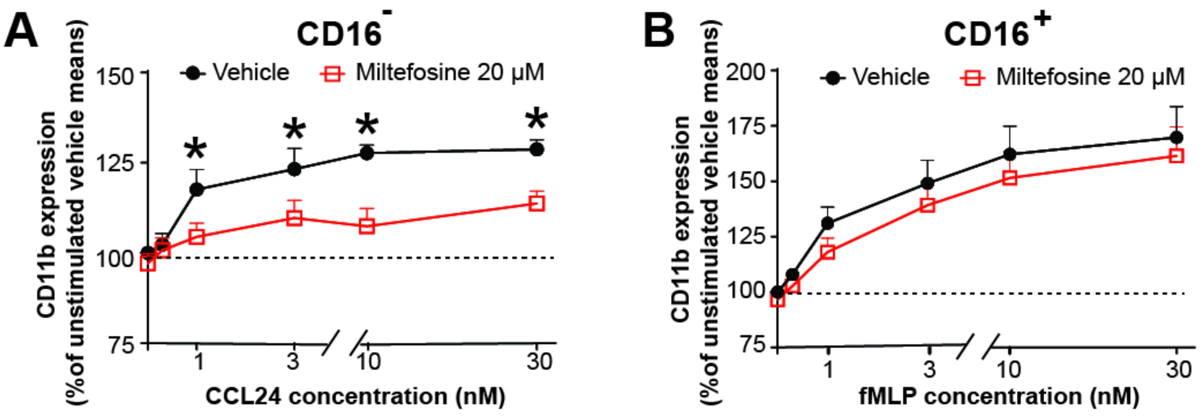

Figure S3. Miltefosine does not inhibit CD11b upregulation on neutrophils. PMNL were stained with anti-CD16 and anti-CD11b and then treated either with miltefosine (20 μM) or vehicle control (15min, RT). (**A**) Cells were stimulated with CCL24 for 4 min at 37°C and CD11b expression was analyzed by flow cytometry on $CD16^-$ cells (eosinophils) and expressed as percent of unstimulated vehicle control. (**B**) Cells were stimulated with fMLP for 4 min at 37°C and CD11b expression was analyzed by flow cytometry on $CD16^+$ cells (neutrophils) and expressed as percent of unstimulated vehicle control. Data are shown as mean + SEM from five individual experiments. *p<0.05, vs vehicle (Two-Way ANOVA with Bonferroni post hoc test).

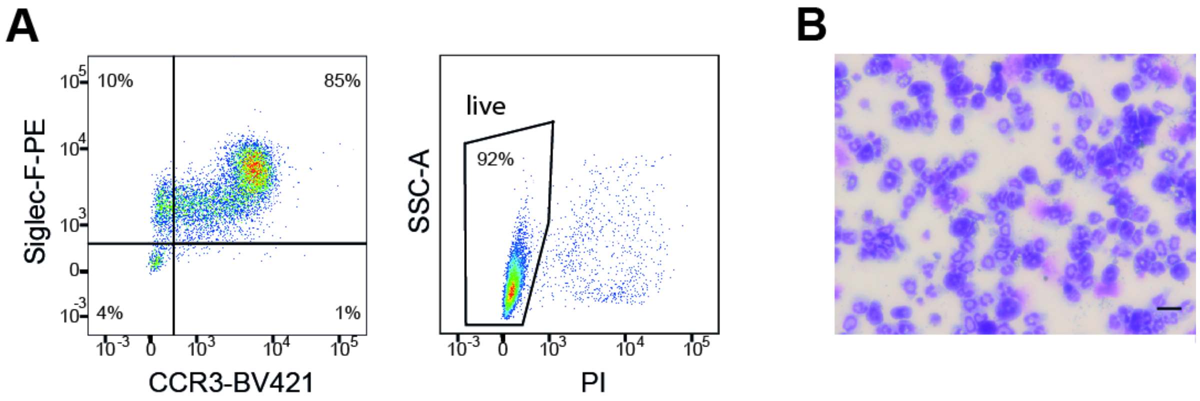

Figure S4: Differentiated bone marrow derived eosinophils (BMDEs) stain positive for mouse eosinophil markers and exhibit typical mouse eosinophil morphology. (**A-B**) BMDEs were differentiated from bone marrow of BALB/c mice for 14 days. (**A**) Purity (Siglec-F.positive, CCR3 positive) and viability (PI negative) was assessed with flow cytometry. Representative plot of eosinophils isolated from one mouse is shown. (**B**) Representative microscopy image of H&E stained cytospin of BMDEs from one mouse on day 14 of the differentiation protocol is shown, calibration bar: 20 µm.

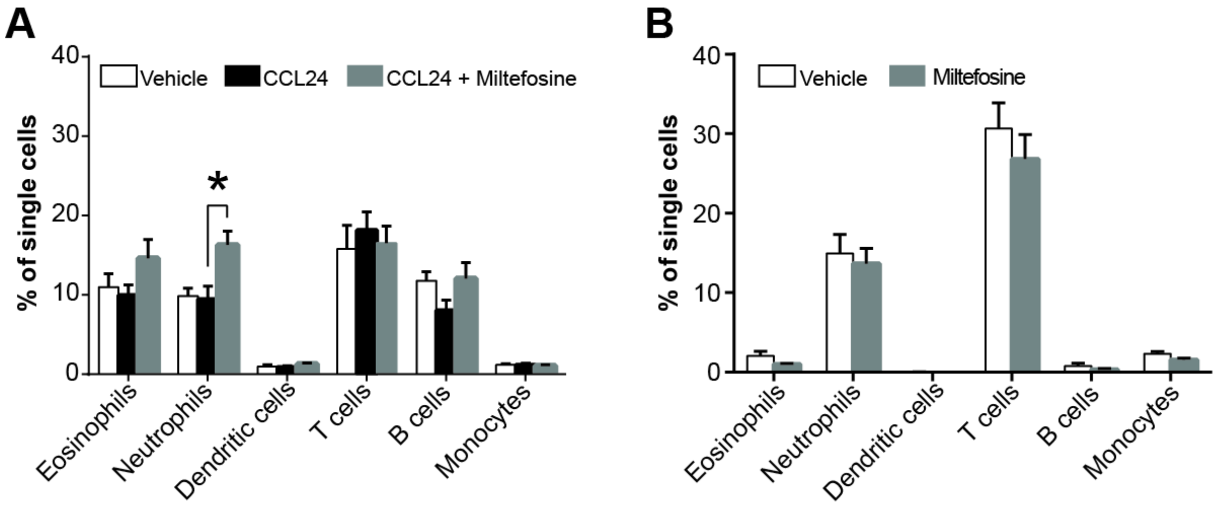

Figure S5. Immune cell composition in blood. Eight week old IL-5Tg **(A)** or BALB/c **(B)** mice received either miltefosine (20 mg/kg) or vehicle (0.9% NaCl) per os for three consecutive days. **(A)** On the last day mice received intranasal application of CCL24 or vehicle. Subsequently after four hours blood was collected and analyzed for immune cell composition. Data are shown as mean + SEM from 6-8 mice from two individual experiments. *p<0.05, vs eotaxin group (One-Way ANOVA with Dunnett´s post hoc test). **(B)** Four hours after last treatment blood was collected and analyzed for immune cell composition. Data are shown as mean + SEM from 10 mice from two individual experiments.

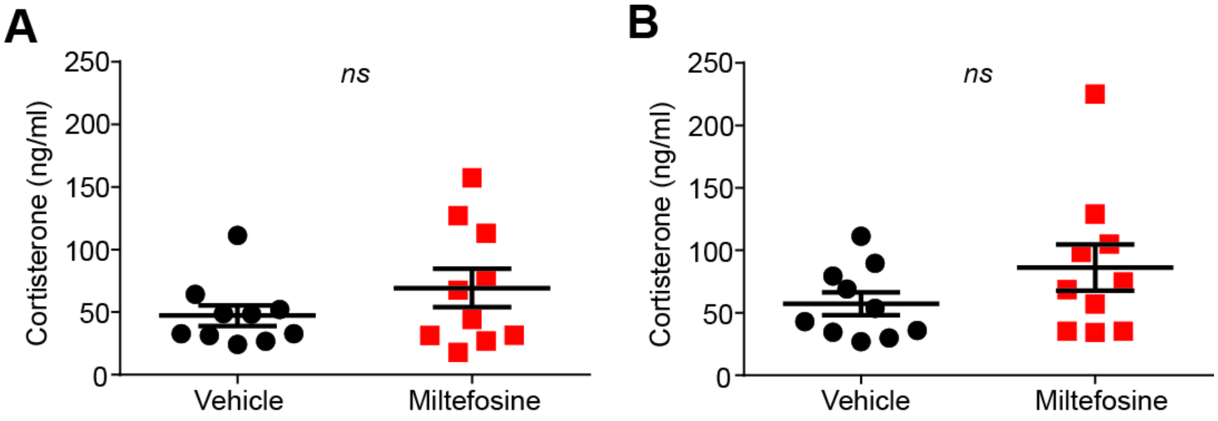

Figure S6. Corticosterone levels in plasma are not significantly altered following miltefosine treatment. (**A-B**) BALB/c mice received either vehicle (0.9% NaCl) or miltefosine (20 mg/kg) per os for three consecutive days. (**A**) Blood was sampled five hours after first treatment on day 1 or four hours following last treatment on day 3 (**B**). Corticosterone levels were assessed with a specific enzyme immunoassay kit. Data are shown as mean ± SEM from 10 mice.

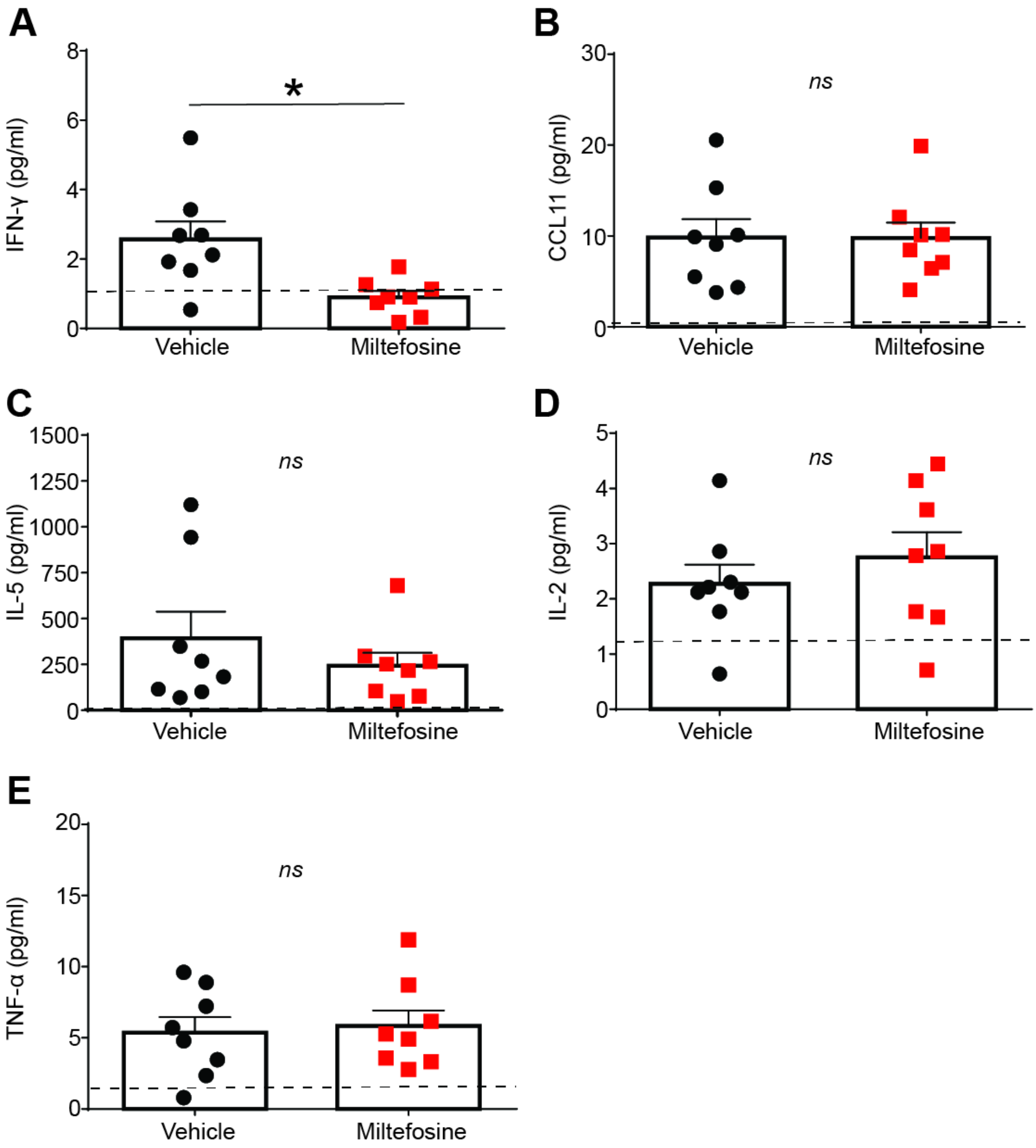

Figure S7: Miltefosine treatment decreases IFN-γ in BAL fluid of BALB/c mice in a model of allergic inflammation. Eight-week-old BALB/c mice were sensitized with ovalbumin intraperitoneal (i.p.) at day 0 and at day 7 and were subsequently treated with miltefosine (20 mg/kg) or vehicle per os daily from day 7 to day 17. Afterwards mice were challenged with an ovalbumin aerosol on day 14 and day 16 followed by sampling of BAL fluid. BAL fluid supernatants were subsequently analysed with a custom multiplex ELISA testing for IFN-γ (**A**), CCL11 (**B**), IL-5 (**C**), IL-2 (**D**) and TNF-α (**E**) levels. Data are shown as mean + SEM from 8 mice per group. Dotted line represents the lower limit of quantification (LLOQ) for each measured cytokine determined from its standard curve. *$p<0.05$ analyzed with Mann-Whitney U test.

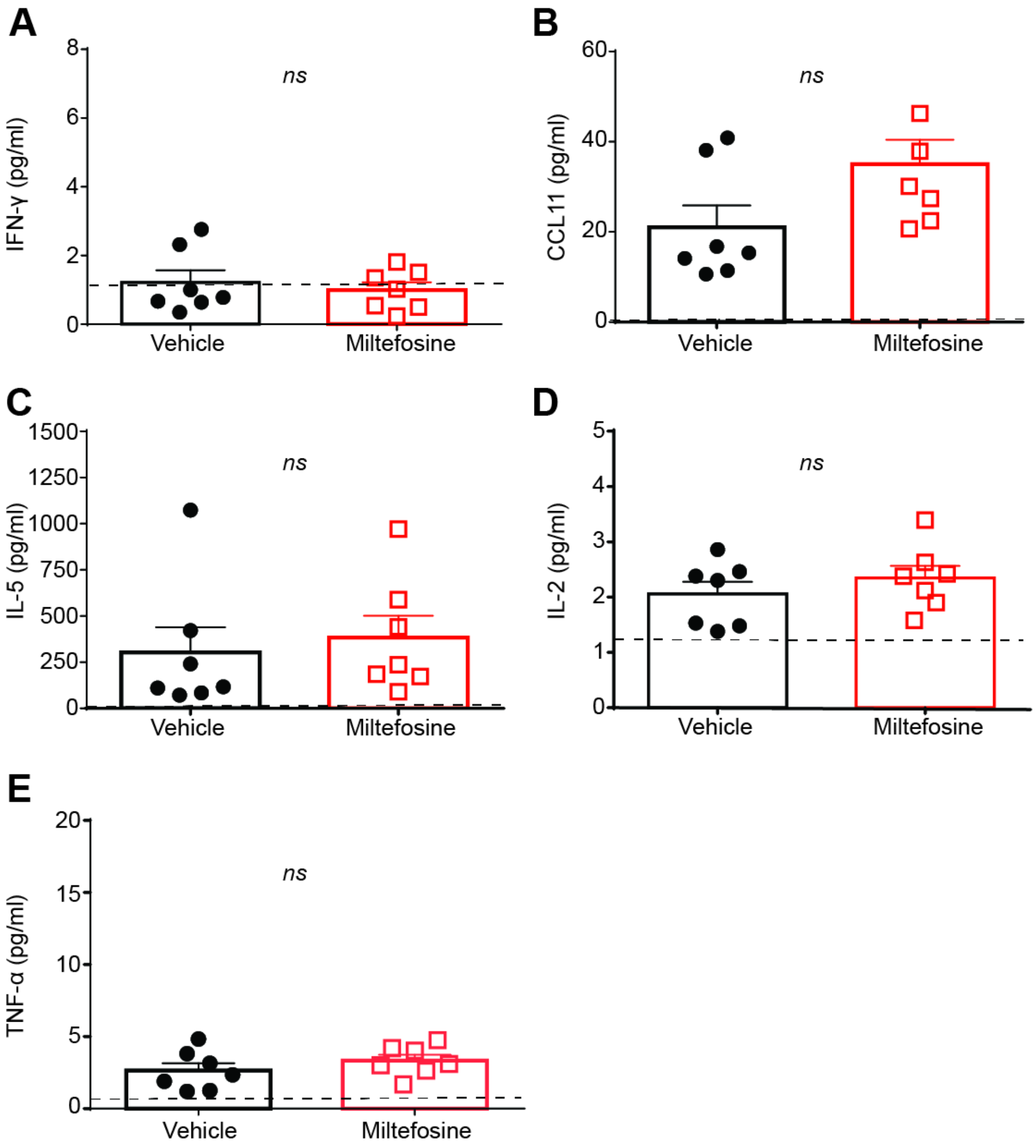

Figure S8: Miltefosine treatment does not alter cytokine content in the BAL fluid of eosinophil deficient mice. Eight-week-old ΔdblGATA-1 mice were sensitized with ovalbumin intraperitoneal (i.p.) at day 0 and at day 7 and were subsequently treated with miltefosine (20 mg/kg) or vehicle per os daily from day 7 to day 17. Afterwards mice were challenged with an ovalbumin aerosol on day 14 and day 16 followed by sampling of BAL fluid. BAL fluid supernatants were subsequently analysed with a custom multiplex ELISA testing for IFN-γ (**A**), CCL11 (**B**), IL-5 (**C**), IL-2 (**D**) and TNF-α (**E**) levels. Data are shown as mean + SEM from 7 mice per group. Dotted line represents the lower limit of quantification (LLOQ) for each measured cytokine determined from its standard curve.
